# Overexpression of *McHB7* Transcription Factor from *Mesembryanthemum crystallinum* Improves Plant Salt Tolerance

**DOI:** 10.3390/ijms23147879

**Published:** 2022-07-17

**Authors:** Xuemei Zhang, Bowen Tan, Zihan Cheng, Dan Zhu, Tingbo Jiang, Sixue Chen

**Affiliations:** 1 State Key Laboratory of Tree Genetics and Breeding, School of Forestry, Northeast Forestry University, Harbin 150040, China; zhangxuemei199111@gmail.com (X.Z.); 15546010865@163.com (Z.C.); 2 Department of Biology, Genetics Institute, University of Florida, Gainesville, FL 32610, USA; tanbowen@ufl.edu (B.T.); zhudan2014dora@163.com (D.Z.); 3 Department of Plant Biology, College of Life Sciences, Qingdao Agricultural University, Qingdao 266109, China; 4 Plant Molecular and Cellular Biology Program, University of Florida, Gainesville, FL 32610, USA; 5 Proteomics and Mass Spectrometry, University of Florida, Gainesville, FL 32610, USA

**Keywords:** *Mesembryanthemum crystallinum*, *Mc*HB7, Arabidopsis, proteomics, metabolomics, salt stress

## Abstract

*Mesembryanthemum crystallinum* (common ice plant) is one of the facultative halophyte plants, and it serves as a model for investigating the molecular mechanisms underlying its salt stress response and tolerance. Here we cloned one of the homeobox transcription factor (TF) genes, *McHB7*, from the ice plant, which has 60% similarity with the Arabidopsis *AtHB7*. Overexpression of the *McHB7* in Arabidopsis (OE) showed that the plants had significantly elevated relative water content (RWC), chlorophyll content, superoxide dismutase (SOD), and peroxidase (POD) activities after salt stress treatment. Our proteomic analysis identified 145 proteins to be significantly changed in abundance, and 66 were exclusively increased in the OE plants compared to the wild type (WT). After salt treatment, 979 and 959 metabolites were significantly increased and decreased, respectively, in the OE plants compared to the WT. The results demonstrate that the *McHB7* can improve photosynthesis, increase the leaf chlorophyll content, and affect the TCA cycle by regulating metabolites (e.g., pyruvate) and proteins (e.g., citrate synthase). Moreover, *Mc*HB7 modulates the expression of stress-related proteins (e.g., superoxide dismutase, dehydroascorbate reductase, and pyrroline-5-carboxylate synthase B) to scavenge reactive oxygen species and enhance plant salt tolerance.

## 1. Introduction

Salt stress has been a major abiotic factor affecting plant growth, development, and productivity due to climate change and modern agricultural practice. It poses various adverse effects, including osmotic stress and ionic stress to plant cells, causing yield loss and global food insecurity [1,2]. Under salt stress, plants manifest a variety of morphological, physiological, and biochemical changes, such as water loss [3], reactive oxygen species (ROS) burst [4], chlorophyll degradation [5], growth quiescence [6], and cell death [7]. In response to salt stress, many plants also exhibit plasticity by initiating acclimation or adaptation processes to protect themselves from the damage and thus increase their tolerance to high-salinity conditions [8]. To date, fundamental mechanisms of plant salt response have been explored, such as salt perception and transport, Ca^2+^ and ROS waves, salt overly sensitive (SOS) pathway activation, transcriptional regulation, phospholipid modification, protein kinase signaling, ABA signaling, auxin transport, metabolite changes, carbon partitioning, and translocation [9,10,11]. In recent years, the molecular mechanisms of salt response have been studied in different species such as Arabidopsis [12], rice [13], wheat [14], and soybean [15]. However, *Mesembryanthemum crystallinum*, as one of well-recognized halophytes that can survive 800 mM NaCl, has rarely been studied in terms of the molecular mechanisms underlying its salt stress response and tolerance [16].

Transcription factors (TFs) have been reported to play a crucial role in plant salt stress responses by activating and/or repressing the expression of salt-related genes [10], e.g., myeloblastosis (*MYB*) [17], ethylene response factor (*ERF*) [18], basic leucine zipper (*bZIP*) [19], *NAM*, *ATAF*, and *CUC* (*NAC*) [20] and homeodomain associated to a leucine zipper (*HD-ZIP*) [21]. They are involved in different signaling pathways during salt stress response and are well-studied. Here we focused on a salt-induced HD-ZIP TF from the ice plant, containing a conserved homeodomain (HD) for DNA binding and a dimerization motif homeobox associated leucine zipper (HALZ) [22]. According to PlantTFDB, 8602 HD-ZIP TFs have been identified in 165 species (http://planttfdb.gao-lab.org/family.php?sp=Ath&fam=HD-ZIP, accessed on 18 March 2022). Among them, *Arabidopsis thaliana* has 58 in total, including 10 redundant TFs. They were divided into four subfamilies according to their structures, conservation of HD-ZIP domain, additional conserved motifs, and functions [23]. However, no genome-wide research and functional studies of the HD-ZIP TFs in ice plant have been reported. In rice, for instance, salt-induced *Oshox22* significantly enhanced tolerance to salt stress through ABA-mediated signal transduction pathways [24]. Overexpression of a *MdHB7* in apple improved its photosynthetic performance and reduced ROS and Na^+^ accumulation under salt stress [25]. A salt-induced *HB1* from *Medicago truncatula* played a role in regulating root architecture and lateral root emergence [26].

Based on our previous transcriptomic study on ice plants under salt stress [27], a *McHB7* was significantly upregulated after 500 mM NaCl treatment for seven days. In another study [28], we cloned the nuclear-localized gene *McHB*7 from the ice plant. Transient expression of the *McHB7* in ice plants effectively improved plant salt tolerance. The result may be attributed to the changes in the activities of stress-related enzymes, such as SOD and POD, and photosynthesis proteins. To further explore the molecular functions of *McHB7* in plant salt stress response, we obtained stable *McHB7*-overexpressing (OE) transgenic Arabidopsis, using the method of *Agrobacterium*-mediated transformation. We first examined the changes of physiological parameters in the OE plants under control and salt stress. Then we conducted proteomics and metabolomics of the OE and wild-type (WT) plants under control and salt stress. The results have provided insightful information about the *Mc*HB7 functions in plant salt stress responses.

## 2. Results

### 2.1. AtHB7 Is the Homolog of McHB7

**Figure 1:**
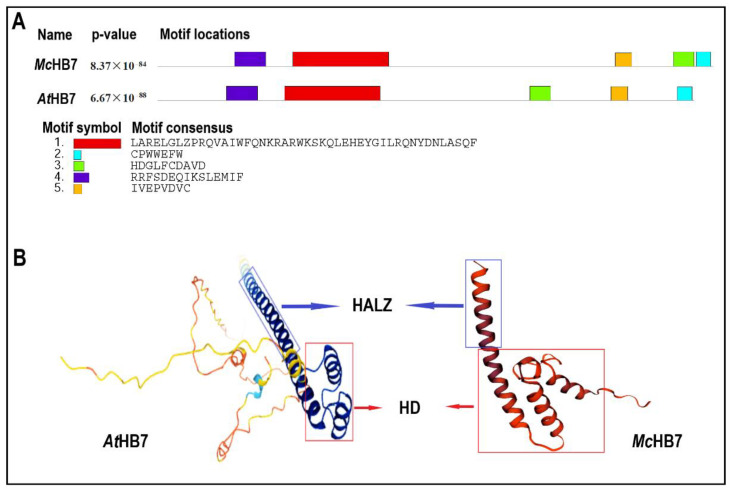
Sequence analysis of *Mc*HB7 and *At*HB7. (**A**) Conserved motifs of *Mc*HB7 and *At*HB7. (**B**) Three-dimensional structure of *At*HB7 and the predicted structure of *Mc*HB7. HD, homeobox domain. HALZ, homeobox-associated leucine zipper.

*McHB7* is 804 bp in length, and it encodes a protein of 267 amino acids. According to NCBI Blast, the homologous Arabidopsis gene *AtHB7* has 60% sequence similarity, with a bitscore of 106.7 and an e-value of 2.3 × 10^−23^. MEME prediction showed that the amino acid sequences of *Mc*HB7 and *At*HB7 have five conserved motifs (Figure 1A). Motif 1 and Motif 2 consist of a typical DNA-binding domain homeodomain (HD). It folds into three alpha-helices (Figure 1B), which are characteristics of all the Homeobox TFs in eukaryotic organisms [23]. Homeobox-associated leucine zipper (HALZ), serving as a dimerization motif, locates immediately downstream of the HD [29]. Motifs 3, 4, and 5 were detected in the carboxy-terminal region of both *Mc*HB7 and *At*HB7, but at different locations (Figure 1A).

### 2.2. Creation of McHB7 Transgenic Arabidopsis 

**Figure 2:**
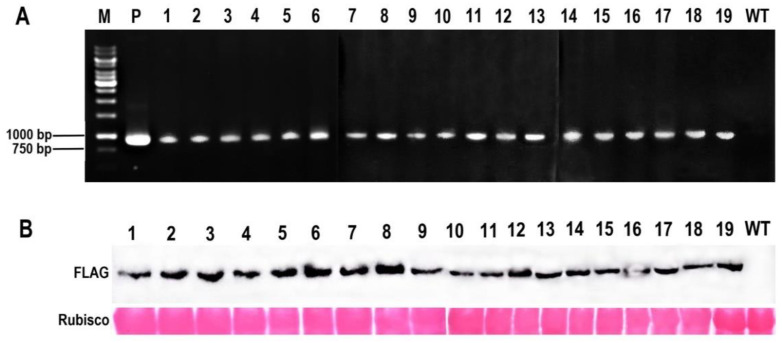
Identification of *McHB7*-overexpressing (OE) transgenic Arabidopsis. (**A**) DNA gel image showing identification of *McHB7* transcripts by PCR. M, 2000 DNA marker. P, positive control, recombinant plasmid. Nos. 1–19, transgenic Arabidopsis lines. WT, wild type Arabidopsis as negative control. (**B**) Western blot image of the *Mc*HB7 protein in the 19 Arabidopsis OE lines.

Using agrobacterium-mediated transformation, a *McHB7-3 × FLAG* construct driven by 35S promoter was introduced to Arabidopsis. At the DNA level, a total of 19 *McHB7* overexpression Arabidopsis (OE) lines were identified. They had the same band at 903 bp as the positive control, which is absent in the WT negative control (Figure 2A). At the protein level, a *Mc*HB7 protein with FLAG tag was observed in all the 19 transgenic OE lines (Figure 2B). These results indicated that *McHB7* has been successfully transformed into the 19 transgenic Arabidopsis lines.

### 2.3. Physiological Characteristics of McHB7-OE Plants under Salt Stress

**Figure 3:**
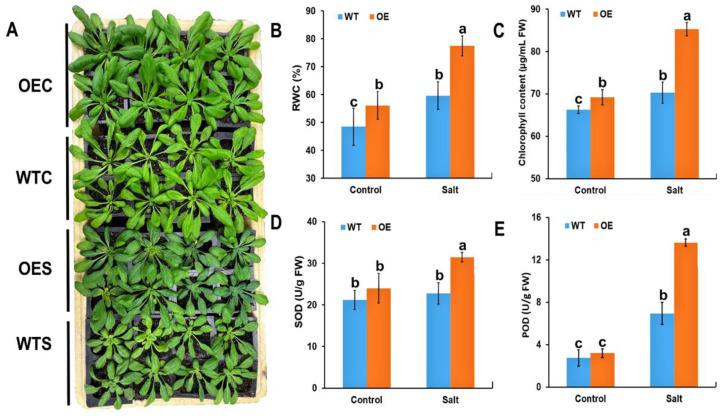
Phenotype and physiological characteristics of the OE and WT plants under control and salt stress conditions. (**A**) Phenotypes of OE and WT under control and salt stress conditions. OEC, *McHB7*-OE Arabidopsis irrigated with water. WTC, wild-type Arabidopsis irrigated with water. OES, *McHB7*-OE Arabidopsis treated with 200 mM NaCl for seven days. WTS, wild-type Arabidopsis treated with 200 mM NaCl for seven days. (**B**) RWC of OE and WT under control and salt stress conditions. (**C**) Chlorophyll content. (**D**) SOD activity. (**E**) POD activity. Error bars indicate mean ± SD (*n* = 4). Means denoted by the same letter did not significantly differ at *p* < 0.05, according to Duncan’s multiple range test.

To explore the function of *Mc*HB7 in response to salt stress, four-week-old OE (line 8) and WT seedlings were irrigated with water and 200 mM NaCl for seven days. As shown in Figure 3A, under control conditions, there is no differences between OE and WT. While the growth of OE is much better than WT after the salt stress treatment. Under salt stress, relative water contents (RWCs) of OE and WT were all elevated, but OE was significantly higher than WT (Figure 3B). The chlorophyll of OE was also significantly increased compared to the WT under control and salt stress conditions. This is consistent with the darker color of OE leaves compared to WT, especially after salt stress treatment (Figure 3C). SOD and POD are considered as important enzymes in ROS removal [30]. In OE plants, the two enzymes exhibited 1.4- and 2-times higher activities than WT under salt stress, respectively (Figure 3D,E). This result suggests that overexpression of the *McHB7* may contribute to enhance plant salt tolerance by improving the activities of stress-related enzymes.

### 2.4. Protein Changes in McHB7-OE Plants under Salt Stress Conditions

**Figure 4:**
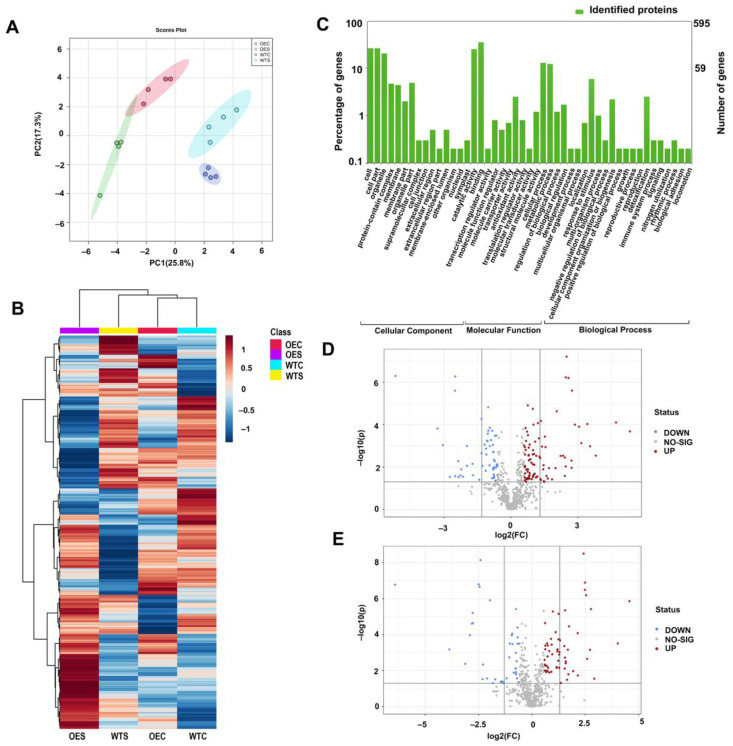
Proteomics analysis of OE and WT under control and salt stress conditions. (**A**) PCA analysis of proteomics data of OEC, WTC, OES, and WTS samples. Four biological replicates were included. (**B**) Clustering of the differential proteins in OEC, WTC, OES, and WTS. (**C**) GO enrichment of the identified proteins, including cellular component, molecular function, and biological process. (**D**) Significantly changed proteins in OE compared to WT under control conditions. (**E**) Significantly changed proteins in OE compared to WT after salt stress treatment.

To further explore the mechanisms of *Mc*HB7 function in response to salt stress at the protein level, we extracted proteins from OE and WT leaf samples under control and salt stress conditions, respectively, and conducted label-free proteomics. Principal component analysis (PCA) showed that the four groups of OEC, WTC, OES, and WTS were clearly separated, and within each group, the four replicates were clustered. PC1 represented the separated groups that had 25.8% variation, and PC2 explained 17.3% of the total variation among the samples (Figure 4A). Using Proteome Discoverer™ 2.5, 595 proteins were identified (Figure 4B and Supplementary Table S1), and these proteins were involved in three GO levels (Figure 4C). Molecular function contains catalytic activity (GO:0003824); binding (GO:0005488), including modified amino acid, small molecule, lipid, ion, protein, and protein-containing complex; transcription regulator activity (GO:0140110); molecular function regulator activity (GO:0098772); antioxidant activity (GO:0016209); translation regulator activity (GO:0045182); and molecular transducer activity (GO:0060089), including the signaling receptor. Biological processes include cellular processes such as the stimulus response (GO:0051716), cellular homeostasis (GO:0019725), signal transduction (GO:0007165), and stomatal movement (GO:0010118); and metabolic processes such as oxidation-reduction (GO:0055114) and hormone metabolic processes (GO:0042445). The cellular component includes cell (GO:0005623) or cell part (GO:0044464), and membrane (GO:0016020) or membrane part (GO:0044425).

Under control conditions, 99 proteins were significantly changed (fold change (FC) > 1.5, *p*-value < 0.05), with 58 increased and 41 decreased (Figure 4D). Among them, some were involved in chlorophyll binding (GO:0016168), photosynthesis (GO:0015979), light harvesting (GO:0009082), and chlorophyll biosynthetic process (GO:0015995). This may explain the dark green color of the OE leaves (Figure 3). Moreover, some proteins were involved in antioxidant activity (GO:0016209), oxidoreductase activity (GO:0016491), regulation of ROS metabolism (GO:2000377), oxidation-reduction process (GO:0055114), and response to abiotic stimulus (GO:0009628). In addition, Q84P23 (AT5G63380.1), a significantly increased protein in the OE plants, was predicted to participate in the jasmonic acid biosynthetic process (GO:0009695), indicating the potential involvement of *Mc*HB7 in hormone signaling.

Under salt stress, 145 proteins were identified to be significantly changed in OE plants compared to WT (FC > 1.5, *p*-value < 0.05), with 90 increased and 55 decreased (Figure 4E). Eighteen of these proteins were annotated to be involved in photosynthesis (GO:0015979), four in chlorophyll biosynthetic process (GO:0015995), eight in pigment biosynthetic process (GO:0046148), and nine in pigment metabolic process (GO:0042440). This result was consistent with the above result the chlorophyll content in OE after the salt stress treatment. Moreover, 11, 26, 40, and 64 proteins take part in the response to oxidative stress (GO:0006979), external stimulus (GO:0009605), stress (GO:0006950), and stimulus (GO:0050896), respectively, indicating that *Mc*HB7 may regulate many stress-related proteins. Interestingly, 66 proteins were exclusively increased in the OE plants after the salt stress treatment (Supplementary Figure S1A). These proteins participate in 10 KEGG pathways (Supplementary Figure S1B), including alanine, aspartate, and glutamate metabolism (ath00250); porphyrin and chlorophyll metabolism (ath00860); pentose phosphate pathway (ath00030); oxidative phosphorylation (ath00190); purine metabolism (ath00230); photosynthesis (ath00195); biosynthesis of amino acids (ath01230); ribosome (ath03010); biosynthesis of secondary metabolites (ath01110); and metabolic pathways (ath01100). The results suggested that, when plants are exposed to salt stress, *Mc*HB7 can positively initiate the synthesis of proteins in different pathways to adjust and adapt to the environment perturbation.

### 2.5. Metabolite Changes in McHB7-OE Plants under Salt Stress Conditions

**Figure 5:**
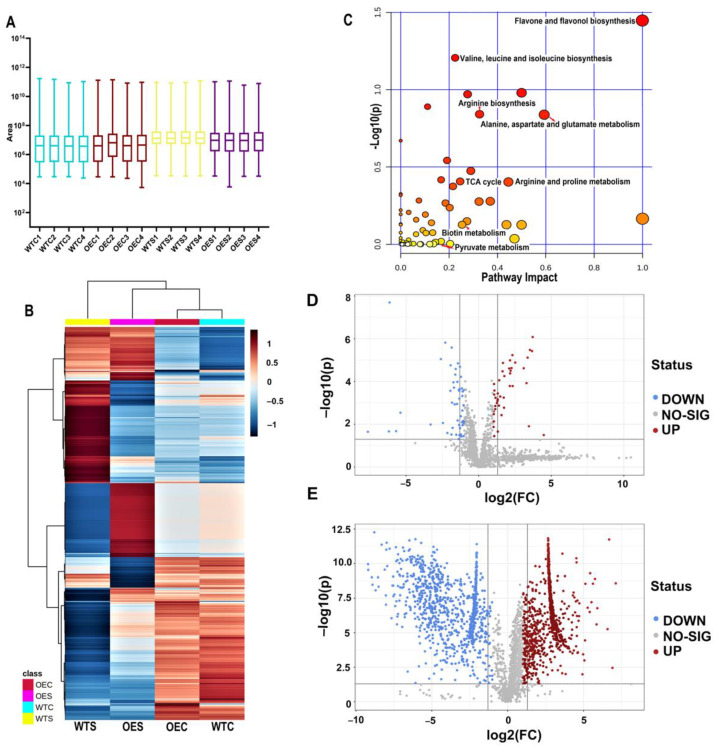
Metabolomic analysis of OE and WT under control and salt stress conditions. (**A**) Box plot showing the median, interquartile range (box), and maximum and minimum scores for each data set. WTC1-4, wild-type Arabidopsis irrigated with water, four biological replicates contained. OEC1-4, *McHB7*-OE Arabidopsis irrigated with water. WTS1-4, wild-type Arabidopsis treated with 200 mM NaCl for seven days. OES1-4, *McHB7*-OE Arabidopsis treated with 200 mM NaCl for seven days. (**B**) Heatmap of identified metabolites in OE and WT under control and salt stress conditions. (**C**) Pathway enrichment of identified metabolites. (**D**) Significantly changed metabolites in OE compared to WT under control conditions. (**E**) Significantly changed metabolites in OE compared to WT after salt stress treatment.

To complement the physiological and proteomic analyses, metabolomics was conducted, using the 16 samples, including OEC, WTC, OES, and WTS, each with four biological replicates. The boxplot showed a discrete distribution of data (Figure 5A). Using Compound Discoverer™ 3.0 Software, a total of 2885 metabolites were identified (Supplementary Table S2). The heatmap in Figure 5B provides a global perspective of the changes of the 2885 metabolites in OE and WT under control and salt stress conditions. A lot of metabolites were predicted to be involved in 65 different pathways, including flavone and flavonol biosynthesis; the TCA cycle; glycolysis/gluconeogenesis; biosynthesis of amino acids (e.g., valine, leucine, isoleucine, arginine, aspartate, and lysine); and metabolism of arginine, glycine, serine, threonine, proline, purine, biotin, ascorbate, pyruvate, and pyrimidine (Figure 5C and Supplementary Table S3). Under control conditions, 42 metabolites were significantly increased and 52 were significantly decreased in the OE plants compared to WT (FC > 2, *p*-value < 0.05; Figure 5D). Among these increased metabolites, some were identified to be involved in ascorbate and aldarate metabolism, purine metabolism, and glucosinolate biosynthesis. In the OE line under salt stress conditions, 979 and 959 metabolites were significantly increased and decreased, respectively (FC > 2, *p*-value < 0.05; Figure 5E). This result suggests that the salt-induced *McHB7* may regulate numerous metabolites when subjected to salt stress. The increased metabolites were found to be involved in 47 KEGG pathways, including the metabolism of pyruvate, ascorbate, and chlorophyll; TCA cycle; glycolysis; and biosynthesis of zeatin, glucosinolate, and flavonoid (Supplementary Table S4).

### 2.6. Integrated Physiological, Proteomics, and Metabolomics of the OE Plants under Salt Stress

**Figure 6:**
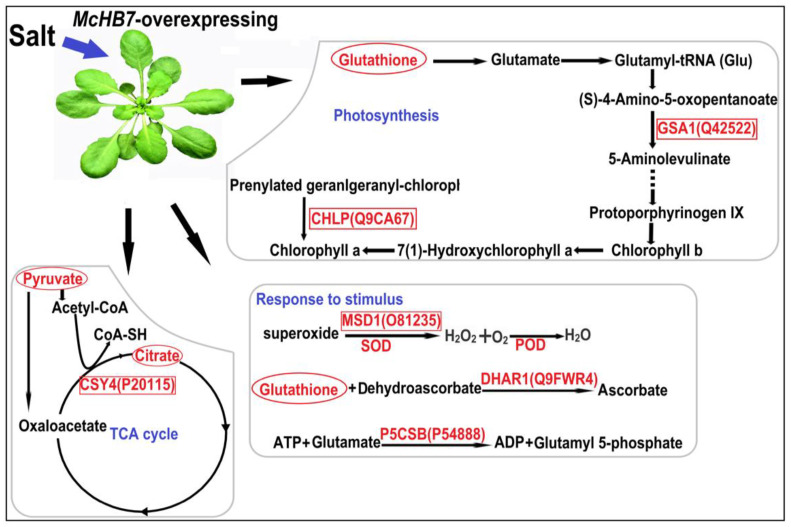
Schematic representation of *Mc*HB7 functions in salt response of the OE Arabidopsis. The words in red represent an increase in the OE plants under salt stress. The words enclosed in a square and oval represent increased proteins and metabolites, respectively. SOD (superoxide dismutase) and POD (peroxidase) activities increased in the OE plants after salt stress treatment.

Based on the increased proteins and metabolites in the OE Arabidopsis after salt stress compared to WT, we made a schematic diagram to integrate the results of physiological, proteomics, and metabolomics and illustrated molecular mechanisms underlying the *McHB7*-mediated plant salt stress response (Figure 6). *McHB7* mainly functions in the pathway of photosynthesis, TCA cycle, and stimulus response. In the process of photosynthesis, *Mc*HB7 may regulate chlorophyll; pyruvate; glutathione; and proteins such as glutamate-1-semialdehyde 2,1-aminomutase 2 (GSA1, Q42522) and geranylgeranyl diphosphate reductase (CHLP, Q9CA67). Our physiological results showed that chlorophyll was increased in the OE plant compared to WT under salt stress, as is consistent with the omics results. Pyruvate, as an important substrate, is involved in various pathways, such as the TCA cycle, amino acid biosynthesis, and nicotinate metabolism. In the TCA cycle, pyruvate can be converted into acetyl-CoA by oxidative conversion. Moreover, oxaloacetate reacting with Acetyl-CoA to generate citrate is also derived from pyruvate. Citrate synthase 4 (CSY4, P20115) catalyzes the reaction of oxaloacetate to citrate, and it keeps the TCA cycle operational to provide energy for plants under the salt stress. In addition, SOD and POD were elevated in OE plants under salt stress compared to WT. They help plants remove excess ROS. Similar to SOD, another significantly increased protein superoxide dismutase 1 (MSD1, O81235) in the OE can function to destroy superoxide anion radicals in mitochondria. Moreover, glutathione S-transferase DHAR1 (Q9FWR4) is a key component of the ascorbate recycling system and is involved in the redox homeostasis. Last but not least, Delta-1-pyrroline-5-carboxylate synthase B (P5CSB, P54888) regulates osmoregulation and maintains the osmotic homeostasis under salt stress conditions.

## 3. Discussion

The ice plant has been gradually known and cultivated because of its stress resilience, fast-growing characteristics, and also edible leaves [31]. Scientifically, ice plant can shift from C_3_ photosynthesis to Crassulacean acid metabolism (CAM) [32], which stimulates CO_2_ fixation and accumulates malic acid in the night [33]. It has been reported that the shift from C_3_ to CAM in the ice plant could be induced by high salinity and decreased soil water content [34]. Since not all the CAM species are salt tolerant [35], the ice plant, as a halophyte, is a useful model for studying plant stress tolerance and enhanced water use efficiency. Considering that soil salinization has become worse and worse due to mineral weathering and irrational use of irrigation and fertilizers [36], the goal of this study was to uncover the molecular mechanisms underlying plant salt stress response and tolerance in the ice plant. The acquired knowledge may inform engineering and breeding efforts for stress-tolerant crops.

HD-ZIP belongs to a large superfamily of homeobox TFs in plants [37]. They are characterized by a highly conserved HD and a variant HALZ that mediates homo- and hetero-dimerization [38]. Similar to its Arabidopsis homolog, *At*HB7, *Mc*HB7 contains the conserved HD and varied locations of motifs in the HALZ domain (Figure 1A). This highlights the similarity and diversity between plants in *Aizoaceae* and *Cruciferae*. The *HD-ZIP* genes are considered as key players in plant growth and development, e.g., controlling the leaf differentiation mechanism, plant architecture, organogenesis, and reproductive processes [39]. Most HD-ZIP TFs play a central role in providing resistance against abiotic stresses, including salt [38], drought [40], heat stress [41], and low temperature [42,43] in different species. Therefore, it is meaningful to uncover the mechanisms of the HD-ZIP regulatory systems in plant response to abiotic stresses. Here we isolated one of *HD-ZIP* gene *McHB7* from the ice plant, which is highly induced by salt stress [27,44], and we focused on its function in response to salinity. To date, little is known about the functions of TFs in the salt stress response of the ice plant, not to mention the HD-ZIP TFs. In this study, *Mc*HB7 was the first HD-ZIP TF functionally characterized for its roles in plant salt stress response.

RWC has been used as one of the important indicators of cell and tissue hydration state [45]. Increased RWC is important to support plant growth under stresses. In our study, RWC increased in *McHB7*-OE under salt stress treatment, suggesting that *McHB7* may prevent plants from losing water even under stress conditions. Moreover, salt stress usually induces a burst of ROS, which could trigger oxidative stress and damage to important biological macromolecules and then lead to cell death [46]. SOD and POD are usually considered as antioxidant enzymes for ROS scavenging, thus contributing to alleviating the harmful effects of ROS such as O_2_^-^ and H_2_O_2_ [47]. *McHB7*-OE plants showed higher activities of SOD and POD under salt stress conditions, as is consistent with the previous study in the ice plant that transgenic plants had significantly higher SOD and POD compared to WT when treated with salt stress [28]. These results suggest that *McHB7* plays a positive role in salt stress response through ROS removal. Meanwhile, based on the proteomics result, some proteins, such as MSD1 (a SOD-like protein), were identified to be increased in the OE plants under salt stress compared to WT. It further supports the *Mc*HB7-mediated improvement of plant salt tolerance through regulating the stress-related enzymes.

Photosynthesis, as the carbon fixation process, provides the primary building blocks and energy for plant growth and development [48]. Under salt stress, plant photosynthesis capability is negatively affected due to decreases in chlorophyll and photosynthetic proteins [39]. In contrast, the *McHB7-OE* plants had significantly elevated chlorophyll contents under control and salt stress conditions, thus maintaining the operation of photosynthesis under salt stress. This is corroborated by the proteomics and metabolomics results. For example, many differential proteins or metabolites in the OE plants are involved in the photosynthesis and biosynthesis of chlorophyll, such as GSA1 and CHLP. Additionally, it was reported that chloroplast is a major ROS production site, and higher concentrations of ROS can damage the membranes, proteins, and especially some photosynthetic enzymes, thereby decreasing CO_2_ fixation, and then affected photosynthesis [49]. However, in the *McHB7-OE* plants, not only were SOD and POD elevated under salt stress conditions, but the stress-related proteins, such as DHAR1 and P5SCB, were significantly induced by salt stress that would help plants remove extra ROS from chloroplasts and keep the photosynthesis stable. Moreover, the salinity-induced degradation of starch that was required for the synthesis of phosphoenolpyruvate (PEP) used in the primary CO_2_ fixation was found and confirmed in chloroplasts of the ice plant [50]. Among the increased metabolites in OE lines after salt stress treatment, some metabolites were found to be involved in starch metabolism, thus providing a new insight into the function of *Mc*HB7 in plant photosynthesis by regulating starch metabolism. All the results suggest that *Mc*HB7 has the same function as *At*HB7 in Arabidopsis in that both promote chlorophyll levels and photosynthesis in mature plants [51]. It is worth mentioning that a lot of research has demonstrated that the proteins belonging to the photosynthesis play pivotal roles in the response to salinity [10]. For example, the increased proteins by salt stress in *Atriplex canescens* were identified to be involved in photosynthetic electron transport and chlorophyll biosynthesis [52]. In sorghum, 51% of identified salt-stress-induced proteins also function in photosynthesis [53]. In our study, many significantly salt-regulated proteins were annotated to be involved in photosynthesis and chlorophyll biosynthetic process. These results are consistent with the previous study on *McHB7* in the ice plant, which showed the salt-induced proteins were related to the light-harvesting complex [28]. All the results point to the conclusion that overexpression of *McHB7* in plants can promote and modulate the expression of photosynthesis-related proteins under salt stress conditions, hence contributing to the growth and development of plants when exposed to adverse environmental conditions.

Protein–protein interactions play vital roles in metabolic regulation, such as metabolic flux, which provides the possibility of direct channeling of metabolites between enzymes [54]. According to the proteomics data, 66 proteins exclusively increased in the *McHB7-OE* plants, and these proteins were involved in diverse pathways such as amino acids’ biosynthesis and metabolism, secondary metabolites’ biosynthesis, and oxidative phosphorylation (Supplementary Figure S1B). This means that *Mc*HB7 mediates the plant response to salinity by playing a role in regulating different pathways. For instance, the TCA cycle is a common metabolic pathway in aerobic organisms, and it links the metabolism of numerous metabolites, proteins, and fats [55]. Moreover, as one of the main pathways in the cellular respiration process, the mitochondrial TCA cycle functions to supply energy and reduce power [56]. It is also closely related to the process of cellular redox homeostasis [56]. Many of the changed proteins and metabolites, such as CSY4, an important synthase for citrate and pyruvate biosynthesis, were increased in the OE plants after salt stress treatment. They are connected to the TCA-cycle-related enzymes and metabolites and thereby provide energy for plant growth and development under adverse environmental conditions. Taken together, *McHB7* mainly functions as a master regulator in regulating plant photosynthesis, TCA cycle, and maintaining redox homeostasis that help the plants in salt stress response and tolerance (Figure 6).

## 4. Materials and Methods

### 4.1. Plant Material and Salt Treatment

*M. crystallinum* (common ice plant) seeds were planted in a growth chamber, at 12 h (26 °C) light/12 h dark (18 °C) cycle. The fully expanded leaves were used for RNA extraction, which was then reverse-transcripted into cDNA for *McHB7* amplification. Arabidopsis plants were grown in another growth chamber, with a temperature of 24 °C, light of 160 μmol/m^2^s, 16/8 h light/dark cycle, and a relative humidity of 65%. For salt stress treatment, four-week-old seedlings with fully expanded leaves were irrigated with 200 mM NaCl solution for seven days. The plants irrigated with water were used as a control.

### 4.2. McHB7 Sequence Analysis

To determine the homolog gene of *McHB7* in Arabidopsis, the amino acid sequence of *Mc*HB7 was blasted by NCBI Protein Blast (https://blast.ncbi.nlm.nih.gov/Blast.cgi?PROGRAM=blastp, accessed on 18 March 2022). To compare the differences between *Mc*HB7 and its homologs in Arabidopsis, the online software MEME (https://meme-suite.org/meme/tools/meme, accessed on 18 March 2022) was used to find the conserved motifs, and SWISS-MODEL (https://swissmodel.expasy.org/, accessed on 18 March 2022) was used to predict the 3D structure of the *Mc*HB7.

### 4.3. Generation of McHB7 Transgenic Arabidopsis Lines

The full length of *McHB7* from the ice plant was cloned with specific primers F1 and R1 (Supplementary Table S5) by PCR. Sanger sequencing was conducted to confirm the fidelity of the *McHB7* sequence. To ligate *McHB7* to the plant binary expression vector pCAMBIA1300 and to facilitate subsequent testing, primers F2 and R2 with *Bam HI* and *Xba* *I*, respectively, were designed, and the reverse primer contained the sequence of 3 × FLAG tag (Supplementary Table S5). The recombinant vector was then transformed into agrobacteria strain GV3101 and cultured in LB liquid medium containing 50 mg/L kanamycin (Kan) and 25 mg/L rifampicin. Based on a *Agrobacterium*-mediated floral dip method [57], the Arabidopsis plants with young flowers were used for infiltration. The seeds from dipped Arabidopsis were collected and screened on 1/2 MS medium with 50 mg/L Kan. Successful transgenic seedlings were then transplanted into soil for T1 seeds.

### 4.4. Identification of Transgenic Arabidopsis by PCR and Western Blot

To further confirm the *McHB7* OE Arabidopsis plants, T1 seeds were selected on 1/2 MS medium with 50 mg/L Kan again. Leaves from the Kan-resistant seedlings were harvested for RNA extraction. The cDNA template and primers F2 and R2 were used for PCR detection, and the recombinant plasmid *McHB7*-pCAMBIA1300 and WT Arabidopsis were used as positive and negative control, respectively. To test the protein levels, 0.1 g fresh leaves of three-week-old Arabidopsis were ground into powder and immersed into 500 μL protein extraction buffer (150 mM NaCl, 50 mM Tri-HCl, 1 mM EDTA, 1% Triton X-100, and 1 × protease cocktail) at 4 °C for 1 h. The supernatant was collected by centrifugation, at the speed of 10,000 rpm for 10 min, and used as crude protein extraction for Western blot, as previously described [28]. WT was used as negative control.

### 4.5. Physiological Measurement of Transgenic Arabidopsis

Four-week-old OE and WT Arabidopsis were treated with 200 mM salt for seven days; OE and WT under control and salt conditions were labeled as OEC, WTC, OES, and WTS, respectively. For relative water content (RWC) measurement, we followed the method of Guan et al. [58], with minor modifications. Briefly, the fresh weight of each shoot was weighed, immersed immediately in distilled water at 4 °C for 24 h to obtain turgid weight, and then placed in an 80 °C oven for 24 h to measure dry weight. The RWC was calculated according to the formula: RWC = [(Fresh Weight − Dry Weight)/(Turgid Weight − Fresh weight)] × 100%. For the analyses of chlorophyll content, SOD, and POD, 0.3 g fresh leaves were used according to previous methods [32,59]. Four biological replicates were used for each sample.

### 4.6. Protein Extraction and Liquid Chromatography–Tandem Mass Spectrometry (LC–MS/MS)

One gram of fresh Arabidopsis leaves was ground into powder and immersed into 3 mL of protein extraction buffer (100 mM Tris-HCl (pH 8.8), 10 mM EDTA, 0.9 M Sucrose, 20 mM 2-Mercaptoethanol, 1 × protease inhibitor cocktail, and 1 mM PMSF), and 3 mL Tris-saturated phenol, at 4 °C, for 1 h. After centrifugation at 15,000× *g* for 15 min, the upper layer was collected and mixed with five times the volume of 0.1 M ammonium acetate in 100% methanol to obtain protein precipitation. The precipitation was washed by 80% acetone twice and 100% acetone one time and then dissolved in 100 µL dissolution buffer (6 M Urea, 1 mM EDTA, 1% SDS, and 50 mM Tris-HCl (pH 8.5)) at 4 °C for 1 h. After centrifugation at 13,000 rpm for 15 min, the supernatant was used for trypsin digestion and LC–MS/MS analysis, as previously described [60]. Four biological replicates were used in this experiment. Proteome Discoverer™ 2.5 (Thermo Fisher Scientific, Bremen, Germany) was used for protein identification and quantification. The online software MetaboAnalyst 5.0 (https://www.metaboanalyst.ca, accessed on 18 March 2022) was used for data visualizing. WEGO 2.0 (https://wego.genomics.cn/, accessed on 18 March 2022) was used for protein ontology annotation. Venn diagram was made by using Venny 2.1.0 (https://bioinfogp.cnb.csic.es/tools/venny/, accessed on 18 March 2022). STRING was used to analyze the proteins’ interaction.

### 4.7. Metabolite Extraction and Metabolomics Analysis

One gram of fresh Arabidopsis leaves was harvested and freeze-dried in a vacuum lyophilizer for 24 h. Then 10 mg of dry leaves was ground into powder and immersed into 0.5 mL solution I (acetonitrile/isopropanol/water = 3:3:2), 0.5 mL solution II (acetonitrile/water = 1:1), and 0.5 mL solution III (80% methanol), respectively. The supernatant was collected by centrifugation at the speed of 13,000 rpm, at 4 °C, for 20 min, and freeze-dried at 4 °C for 6 h. Two standards, 100 μM lidocaine and 100 μM (1S)-(+)-10-Camphorsulfonic, were added as positive and negative mode internal references, respectively. The metabolite samples were dissolved in 100 μL 3% methanol containing 0.1% formid acid. The supernatant was used for untargeted metabolomic analysis on an Orbitrap Tribrid mass spectrometer connected with a Vanquish™ UHPLC liquid chromatography (Thermo Fisher Scientific, San Jose, CA, USA). For samples running, Accucore C18 columns were used, the pump flow rate was 0.4 mL/min, and the LC gradient procedure was as followed: 0 min, 100% solvent A (0.1% formid acid); 21 min, 40% solvent B (0.1% formid acid and 99.9% acetonitrile); 23 min, 95% solvent B; 24 min, 95% solvent B; 25 min, 0% solvent B; 30 min stop run. Compound Discoverer™ 3.0 Software was used for metabolites’ identification and quantification. Metabolites were identified by searching the mzCloud spectra database and annotated by ChemSpider, Pathway mapping to KEGG, and Metabolika pathways for functional analysis.

### 4.8. Statistical Analysis

Statistical analysis was conducted by using SPSS software (released in 2020, IBM SPSS Statistics for Windows, Version 27.0. Armonk, NY, USA: IBM Corp, USA). Means denoted by different letters indicate significant differences at *p* < 0.05, according to Duncan’s multiple range test.

## 5. Conclusions

In this study, we cloned a homeobox gene, *McHB7*, from ice plant leaves that was significantly induced by salt stress. Its structure was analyzed based on the homolog gene *AtHB7* in Arabidopsis. The *McHB7-OE* plants showed significantly improved RWC and chlorophyll contents and enhanced activities of SOD and POD after salt stress treatment. Moreover, *McHB7* regulates plant photosynthesis based on the physiological, proteomics, and metabolomics results. Moreover, *McHB7* was involved in the TCA cycle regulation, which ensures adequate ATP and reductants for plant growth and development under salt stress conditions. Last but not the least, *Mc*HB7 was involved in regulating the activities and/or levels of SOD, POD, P5CSB, and DHAR1 to alleviate ROS damage. All of these data point to the important function of *Mc*HB7 as a master regulator in plant salt stress response.

## Data Availability

The proteomics data have been deposited to the ProteomeXchange Consortium via the PRIDE partner repository with the dataset identifier PXD033886 (userID: reviewer_pxd033886@ebi.ac.uk; password: BzTxpLYf), the metabolomics data have been deposited to the MetaboLights data repository with the data set identifier MTBLS4860.

## Supplementary Materials

The following supporting information can be downloaded at: https://www.mdpi.com/article/10.3390/ijms23147879/s1.
