# Supplementary material for: Overexpression of McHB7 Transcription Factor from Mesembryanthemum crystallinum Improves Plant Salt Tolerance
Source: Int J Mol Sci. 2022 Jul 17;23(14):7879. doi: 10.3390/ijms23147879 (PMC9318261; doi:10.3390/ijms23147879)
Supplement: Supplementary file 1 [file ijms-23-07879-s001.zip › ijms-1751701-supplementary.pdf]

Supplementary Files

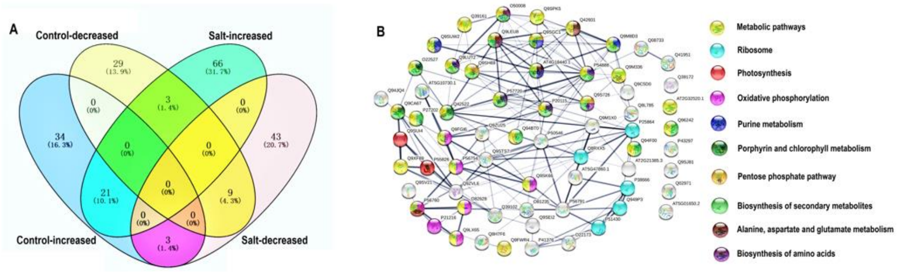

Supplementary Figure S1. Significantly change proteins in the OE compared to WT Arabidopsis plants. **(A)** Venn diagram of identified significantly changed proteins in the OE under control and salt stress conditions including increased and decreased proteins. **(B)** PPI prediction of significantly increased proteins in OE after salt stress by STRING.

Supplementary Table S1. List of proteins identified and quantified in OE and WT plants under control and salt stress conditions

(Please see data in Excel file format)

Supplementary Table S2. List of metabolites identified and quantified in OE and WT plants under control and salt stress conditions

(Please see data in Excel file format)

Supplementary Table S3. KEGG pathway prediction of identified metabolites

KEGG pathway	Total metabolites	Expected	Hits	Raw p	Impact
Flavone and flavonol biosynthesis	10	2.0499	5	0.03569	1
Valine, leucine and isoleucine biosynthesis	22	4.5098	8	0.062216	0.22526
C5-Branched dibasic acid metabolism	6	1.2299	3	0.10469	0.5
Nicotinate and nicotinamide metabolism	13	2.6649	5	0.10702	0.27676
Aminoacyl-tRNA biosynthesis	46	9.4295	13	0.12875	0.11111
Arginine biosynthesis	18	3.6898	6	0.14412	0.32622
Alanine, aspartate and glutamate metabolism	22	4.5098	7	0.1451	0.59352
Monobactam biosynthesis	8	1.6399	3	0.21374	0
Phenylalanine, tyrosine and tryptophan biosynthesis	22	4.5098	6	0.28674	0.19169
Phenylpropanoid biosynthesis	46	9.4295	11	0.33521	0.28998
Glyoxylate and dicarboxylate metabolism	29	5.9447	7	0.38256	0.16723
Citrate cycle (TCA cycle)	20	4.0998	5	0.39203	0.24597
Arginine and proline metabolism	34	6.9697	8	0.3951	0.4455
Tyrosine metabolism	16	3.2798	4	0.42193	0.21622
Butanoate metabolism	17	3.4848	4	0.47234	0
Biosynthesis of unsaturated fatty acids	22	4.5098	5	0.48119	0
beta-Alanine metabolism	18	3.6898	4	0.52102	0.0754
Tryptophan metabolism	28	5.7397	6	0.52688	0.37037
Glycine, serine and threonine metabolism	33	6.7647	7	0.52933	0.3242
Purine metabolism	63	12.914	13	0.54115	0.18536
Lysine biosynthesis	9	1.8449	2	0.57936	0.2027
Indole alkaloid biosynthesis	4	0.81996	1	0.60094	0
Sulfur metabolism	15	3.0748	3	0.62154	0.03315
Caffeine metabolism	10	2.0499	2	0.63991	0
Glutathione metabolism	26	5.3297	5	0.64229	0.10211
Biosynthesis of secondary metabolites - unclassified	5	1.0249	1	0.68297	1
Vitamin B6 metabolism	11	2.2549	2	0.6934	0.0641
Sphingolipid metabolism	17	3.4848	3	0.70945	0.27404
Pantothenate and CoA biosynthesis	23	4.7148	4	0.7254	0.12743
Nitrogen metabolism	12	2.4599	2	0.74016	0
Lysine degradation	18	3.6898	3	0.74713	0
Biotin metabolism	18	3.6898	3	0.74713	0.25385
Cutin, suberine and wax biosynthesis	18	3.6898	3	0.74713	0.4375
Isoquinoline alkaloid biosynthesis	6	1.2299	1	0.74817	0.5
Glucosinolate biosynthesis	65	13.324	11	0.811	0.09164
Tropane, piperidine and pyridine alkaloid biosynthesis	8	1.6399	1	0.8412	0

Stilbenoid, diarylheptanoid and gingerol biosynthesis	8	1.6399	1	0.8412	0.13235
Histidine metabolism	15	3.0748	2	0.84546	0.10465
Thiamine metabolism	22	4.5098	3	0.86029	0.06358
Ascorbate and aldarate metabolism	18	3.6898	2	0.91054	0
Phenylalanine metabolism	11	2.2549	1	0.92058	0.47059
Propanoate metabolism	20	4.0998	2	0.93859	0.03259
Zeatin biosynthesis	21	4.3048	2	0.94927	0
Pyruvate metabolism	22	4.5098	2	0.95817	0.16731
Valine, leucine and isoleucine degradation	37	7.5846	4	0.963	0.00991
Pyrimidine metabolism	38	7.7896	4	0.9685	0.14389
Flavonoid biosynthesis	47	9.6345	5	0.97769	0.02471
alpha-Linolenic acid metabolism	28	5.7397	2	0.98726	0
Cyanoamino acid metabolism	29	5.9447	2	0.9896	0
Terpenoid backbone biosynthesis	30	6.1497	2	0.99151	0.05267
Cysteine and methionine metabolism	46	9.4295	4	0.99182	0.20463
Glycerolipid metabolism	21	4.3048	1	0.99221	0.00426
Carbon fixation in photosynthetic organisms	21	4.3048	1	0.99221	0.03607
Starch and sucrose metabolism	22	4.5098	1	0.99383	0.13619
Fatty acid elongation	23	4.7148	1	0.99511	0
Glycolysis / Gluconeogenesis	26	5.3297	1	0.99757	0.12036
Glycerophospholipid metabolism	37	7.5846	2	0.99802	0.08348
Folate biosynthesis	27	5.5347	1	0.99808	0.02624
Galactose metabolism	27	5.5347	1	0.99808	0.07998
Ubiquinone and other terpenoid-quinone biosynthesis	38	7.7896	2	0.99839	0.00097
Inositol phosphate metabolism	28	5.7397	1	0.99848	0
Fatty acid biosynthesis	56	11.479	3	0.99974	0.01123
Porphyrin and chlorophyll metabolism	48	9.8395	2	0.99981	0.02926
Fatty acid degradation	37	7.5846	1	0.99982	0
Carotenoid biosynthesis	43	8.8146	1	0.99996	0.00632

Supplementary Table S4. KEGG pathway prediction of increased metabolites.

	Total metabolites	Expected	Hits	Raw p	-log10(p)	Impact
Purine metabolism	63	4.4606	9	0.029644	1.5281	0.17173
Aminoacyl-tRNA biosynthesis	46	3.2569	6	0.10105	0.99544	0.11111
Lysine degradation	18	1.2744	3	0.12964	0.88726	0
Arginine biosynthesis	18	1.2744	3	0.12964	0.88726	0.18641
Glyoxylate and dicarboxylate metabolism	29	2.0533	4	0.1441	0.84134	0.0781
Alanine, aspartate and glutamate metabolism	22	1.5577	3	0.20038	0.69815	0.51798
Nitrogen metabolism	12	0.84963	2	0.20657	0.68493	0
Nicotinate and nicotinamide metabolism	13	0.92043	2	0.23348	0.63176	0.0202
Glutathione metabolism	26	1.8409	3	0.27804	0.5559	0.05046
Tyrosine metabolism	16	1.1328	2	0.31477	0.50201	0
Tryptophan metabolism	28	1.9825	3	0.31803	0.49753	0.01852
Butanoate metabolism	17	1.2036	2	0.34159	0.4665	0
C5-Branched dibasic acid metabolism	6	0.42481	1	0.35685	0.44752	0
Ascorbate and aldarate metabolism	18	1.2744	2	0.36808	0.43406	0
beta-Alanine metabolism	18	1.2744	2	0.36808	0.43406	0.0754
Glycine, serine and threonine metabolism	33	2.3365	3	0.41764	0.3792	0.30168
Citrate cycle (TCA cycle)	20	1.416	2	0.41975	0.37701	0.11571
Monobactam biosynthesis	8	0.56642	1	0.44507	0.35157	0
Thiamine metabolism	22	1.5577	2	0.46924	0.32861	0.06358
Valine, leucine and isoleucine biosynthesis	22	1.5577	2	0.46924	0.32861	0.10727
Lysine biosynthesis	9	0.63722	1	0.48457	0.31464	0
Pantothenate and CoA biosynthesis	23	1.6285	2	0.49304	0.30712	0.12743
Caffeine metabolism	10	0.70802	1	0.52129	0.28292	0
Flavone and flavonol biosynthesis	10	0.70802	1	0.52129	0.28292	0.35
Vitamin B6 metabolism	11	0.77883	1	0.55541	0.25538	0.03205
Riboflavin metabolism	11	0.77883	1	0.55541	0.25538	0.06667
Cysteine and methionine metabolism	46	3.2569	3	0.64523	0.19029	0.13971
Histidine metabolism	15	1.062	1	0.66943	0.17429	0
Sulfur metabolism	15	1.062	1	0.66943	0.17429	0
Arginine and proline metabolism	34	2.4073	2	0.70775	0.15012	0.1371
Sphingolipid metabolism	17	1.2036	1	0.71504	0.14567	0
Zeatin biosynthesis	21	1.4869	1	0.78839	0.10326	0
Carbon fixation in photosynthetic organisms	21	1.4869	1	0.78839	0.10326	0.03607
Phenylalanine, tyrosine and tryptophan biosynthesis	22	1.5577	1	0.80359	0.094967	0.09009

Starch and sucrose metabolism	22	1.5577	1	0.80359	0.094967	0.10163
Pyruvate metabolism	22	1.5577	1	0.80359	0.094967	0.16731
Glucosinolate biosynthesis	65	4.6022	3	0.85358	0.068756	0.08933
Glycolysis / Gluconeogenesis	26	1.8409	1	0.8543	0.068391	0.12036
Flavonoid biosynthesis	47	3.3277	2	0.85908	0.065967	0.00087
Folate biosynthesis	27	1.9117	1	0.8648	0.063086	0.02624
Galactose metabolism	27	1.9117	1	0.8648	0.063086	0.07998
alpha-Linolenic acid metabolism	28	1.9825	1	0.87455	0.058217	0
Cyanoamino acid metabolism	29	2.0533	1	0.8836	0.053744	0
Terpenoid backbone biosynthesis	30	2.1241	1	0.89201	0.049632	0
Glycerophospholipid metabolism	37	2.6197	1	0.93619	0.028635	0.05273
Pyrimidine metabolism	38	2.6905	1	0.94082	0.026491	0
Porphyrin and chlorophyll metabolism	48	3.3985	1	0.97224	0.012227	0

Supplementary Table S5. Information of primers used in this study.

Primer Name	Primer sequence (5’-3’)	Purpose
F1	ATGATGTATGAAGAAGGAGAA	For the cloning of *McHB7*
R1	CGACCAAAATTCCCACCATTG	
F2	CGCGGATCCATGATGTATGAAGAAGGAGAA	To ligate *McHB7* to the plant binary expression vector *pCAMBIA1300*
R2	CGGTCTAGATCAGGCGCCTTTGTCATCGTCATCCTTGTAGTCTCCGCCTTTATCGTCATCGTCTTTATAATCTCCGCCTTTGTCATCGTCATCCTTGTAGTCTCCGCCCGACCAAAATTCCCACCATTG	
